# Transferring the sandwich principle to instructional videos: is it worth the effort?

**DOI:** 10.1186/s12909-021-02967-3

**Published:** 2021-10-09

**Authors:** Anna Bock, Christina Thomas, Marius Heitzer, Philipp Winnand, Florian Peters, Martin Lemos, Frank Hölzle, Ali Modabber

**Affiliations:** 1 Department of Oral and Maxillofacial Surgery, University Hospital RWTH Aachen, Pauwelsstrasse 30, 52074 Aachen, Germany; 2 Audiovisual Media Center, Medical Faculty, RWTH Aachen University, Pauwelsstraße 30, 52074 Aachen, Germany

**Keywords:** Education, Sandwich principle, Instructional video, Activating elements, Educational model

## Abstract

**Background:**

The sandwich principle is an educational concept that regularly alternates between collective and individual learning phases within one learning unit. Applying sandwich principle to lectures has proven to be more effective for learning outcomes than classical lectures. Supposedly, this teaching format also leads to a beneficial knowledge transfer when applied to other teaching formats. Therefore, the aim of this study was to investigate the effect of the sandwich principle on instructional videos and how its use was evaluated by students.

**Methods:**

Participants (*n* = 51) were randomly allocated into two groups. Both groups were given a test to assess the baseline level of knowledge. Afterwards, the control group watched the normal instructional video on cleft lips and palates, while the sandwich group watched the same video modified according to the sandwich principle. The participants then had to answer 30 single-choice questions to assess their knowledge gain and evaluate the instructional video. Long-term retention of the knowledge was tested again 6 months later using the same test questions. The unpaired t-test and ANOVA were used to compare the results.

**Results:**

Comparison of the pre-test and post-test results of both groups showed significantly increased test scores (*p* < 0.0001). Regarding long-term retention, the mean test scores were still significantly higher in both groups than before watching the video (*p* < 0.0001). For all test results, there was no significant difference between the groups (*p* > 0.05). The evaluation showed that the students highly appreciated the modified video and found the interruptions for repetition of previously learned knowledge useful.

**Conclusion:**

The hypothesis that the modification of instructional videos according to the sandwich principle would lead to an improved learning outcome could not be proved subjectively or objectively. Nevertheless, the teaching format was highly appreciated by the students and may have increased their motivation to learn with instructional videos.

**Supplementary Information:**

The online version contains supplementary material available at 10.1186/s12909-021-02967-3.

## Background

For several decades, teacher-centred methods of education have been used in medical education. The most traditional teacher-centred format is face-to-face teaching. It is defined as an instructional method in which a person teaches a group of students, for example, in a lecture hall. Although live interaction occurs between the learners and teacher, the teacher asserts control over the content and the way it is studied by the students. Generally, face-to-face teaching is set to a specific date and time [1].

.However, student-centred learning approaches have increasingly been focused upon in medical education. Student-centred learning provides students autonomy and addresses individual learning needs and styles [2]. It includes a wide variety of instructional approaches and is often associated with learning experiences that occur outside of traditional classroom settings. One medium for supporting student-centred learning is digital learning. Digital learning, or e-learning, can be described as a set of technology-mediated methods [3]. One of these methods often used in medical education is the instructional video. These videos are supposed to increase program effectiveness and student satisfaction [4–6]. Due to the recent pandemic, digital teaching, especially instructional videos, have quickly gained prominence.

A teaching format that centres students in classical settings is called the sandwich principle. The sandwich principle is an educational concept that regularly alternates between collective and individual learning phases within one learning unit. During collective learning phases, students learn passively (i.e., by listening to the lecturer). This phase is supposed to be a compact mediation of knowledge with a maximum duration of 20-25 min, a timeframe that considers the length of the students’ attention span [7, 8]. During the individual learning phases, the students learn actively, through precise work assignments. Therefore, the previously gained knowledge is accessed by repetition or application. For example, activating elements can be small-group work or the basis for partner discussions. Due to a wide variety of activating elements, this phase is supposed to accommodate for learning types and personal preferences. The individual learning phases can also be seen as medical pauses, an interruption of a procedure for a certain period of time [9]. It has been shown that this promotes learning by facilitating the processing and recapping of the previously learned [9, 10]. The use of the sandwich principle has been promoted as a tool for high-quality education [7, 11]. In general, the sandwich principle can be applied to seminars, bedside teaching and classical lectures [7]. In lectures its application has been proven to be more effective and lead to a significantly better learning outcome compared to classical lectures [12]. However, whether applying the sandwich principle to other teaching formats leads to a beneficial knowledge transfer has not been confirmed scientifically. Therefore, the aim of the study was to investigate whether the application of the sandwich principle to instructional videos led to improved knowledge gain and how its use was evaluated by students.

## Methods

### Participants

All fourth-year medical students (*n* = 252) were invited to voluntarily participate in this study. According to the curriculum, the students had no prior knowledge of the topic. The number of participants was calculated based on similar studies [8, 12].

### Study design

The study occurred in the computer lab of the medical faculty to provide a workplace with a headset for each participant. A tutor was always present to provide supervision and help in case of technical problems. Written informed consent was obtained. The participants were randomly allocated into two groups by automatic programming of the e-learning tool. Both groups started with a test to assess their baseline level of knowledge. Afterwards, the control group (group A) watched the normal instructional video, while the sandwich group (group B) watched the same instructional video modified with activating elements according to the sandwich principle. Immediately after watching the video, participants had to answer 30 single-choice questions to assess their knowledge gain. Additionally, the tutorial video was evaluated. Long-term retention of the knowledge was tested again 6 months later using the same test questions. The need for ethics approval was waived by the institutional review board (EK 137/15). All methods were carried out in accordance with relevant guidelines and regulations for data protection.

### Instructional video

The topic of the instructional video was the aetiology and therapy of cleft lips and palates. The video covered incidence and aetiology, embryogenesis, classification, naso-alveolar-moulding-therapy, primary and secondary surgical therapy, and follow-up care. Overall, the video had a duration of 45 min, equal to the duration of a standard lecture or seminar.

### Activating elements

The instructional video modified according to the sandwich principle has two interruptions for the activating elements. The first interruption is at 10 min 18 s, and the second interruption is at 30 min 28 s. As activating elements, five tasks concerning the previously taught content had to be solved, including exercises such as drag-drop, matching, fill-in-the-blank, true/false and short answer questions. After the exercises were edited, participants were immediately informed about the correct answer.

### Learning phase

The duration of the control group’s learning phase was exactly as long as the instructional video. The sandwich group additionally had to edit the activating elements and therefore, their learning phase was about 10 min longer.

### Test

Initially, the learning objectives for the e-learning programme were defined according to the SMART (specific, measurable, assignable, realistic, time-related) criteria [13]. A total of 50 questions based on the learning objectives were generated. Only type A questions were used. These questions consist of five statements with only one correct option [14]. To assess the difficulty level of the questions, these were validated prior to the main study. The difficulty level refers to the group being tested and is calculated on the basis of the reached mean score for the particular question. Difficult questions have a high (0.8 -1) difficulty level and easy questions a low (0.1 - 0.4) difficulty level [15]. For the validation, 20 dental students were recruited, half of which had prior knowledge on the topic ‘cleft lip and palate’ and the other 10 did not. These 20 volunteers were not involved in the main study. The validation of the question catalogue was conducted to eliminate too easy (difficulty level 1) and too difficult (difficulty level 0) test questions. According to the difficulty level, 30 questions were chosen for the main study. The distribution of the difficulty level of the question catalogue and the test are shown in Table 1. Ideally, 60% of the questions have a difficulty level between 0.4 and 0.8, 20% have a lower difficulty level and 20% a higher difficulty level. In evaluating the test, each correct answer received 1 point; there were no half or minus points. The maximum score of the test was 30 points. The same test with a different order of questions and answers was used as pretest, posttest and to assess the long-term retention.

**Table 1:** Distribution of the difficulty level of the question catalogue and the test

Difficulty Level	Question catalogue (***n*** = 50)	Test questions (***n*** = 30)
1	3	0
≥0.8-0.9	6	6
> 0.4- < 0.8	14	14
≤0.4-0.1	26	10
0	1	0

### Evaluation

Both groups evaluated the instructional video and carried out a self-assessment of knowledge acquisition before and after watching the video. Additionally, the sandwich group evaluated the activating elements in terms of usefulness, concentration, reflection of learning content, subjective difficulty level and future use. All aspects were evaluated using a 10-point Likert scale, where 1 denoted ‘fully agree/very good/too easy/appropriate’ and 10 ‘totally disagree/unsatisfactory/too difficult/inappropriate’ (see supplementary material).

### Statistics

The obtained data were arranged using MS Office Excel 2019® (Microsoft Corporation, Redmond, Washington, USA). Statistical analyses were performed using GraphPad Prism 6 Software (GraphPad Software, San Diego, California, USA). All results were checked on normal distribution using the Anderson-Darling normality test. An unpaired t-Test was used to compare the results of the test within the groups and a 2 × 3-factorial ANOVA was used for comparison between the groups. For analyzing the results of the evaluation an unpaired t-test and the Mann-Withney-U test were used. *P* ≤ 0.05 was considered significant. The effect size for discriminating between groups was estimated using Cohen’s *d* effect size and represented as *d* in the Results section. Values were defined as small (0.20–0.49), medium (0.50–0.79), large (0.80–1.29), and very large (above 1.30) [16]. Besides that, Morris modification of the effect size (d_ppc2_) was used to investigate the long-term effect [17].

## Results

### Participants

All participants (*n* = 51, female 34, male 13, n.a. 4) were allocated into two groups, the control group (group A, *n* = 25) or the sandwich group (group B, *n* = 26). 19 participants were 19-22 years old, 15 were 23-26 years, 10 were 27 to 30 years and 4 were older than 30 years. 3 participants did not answer the question about their age.

### Test

**Fig. 1:**
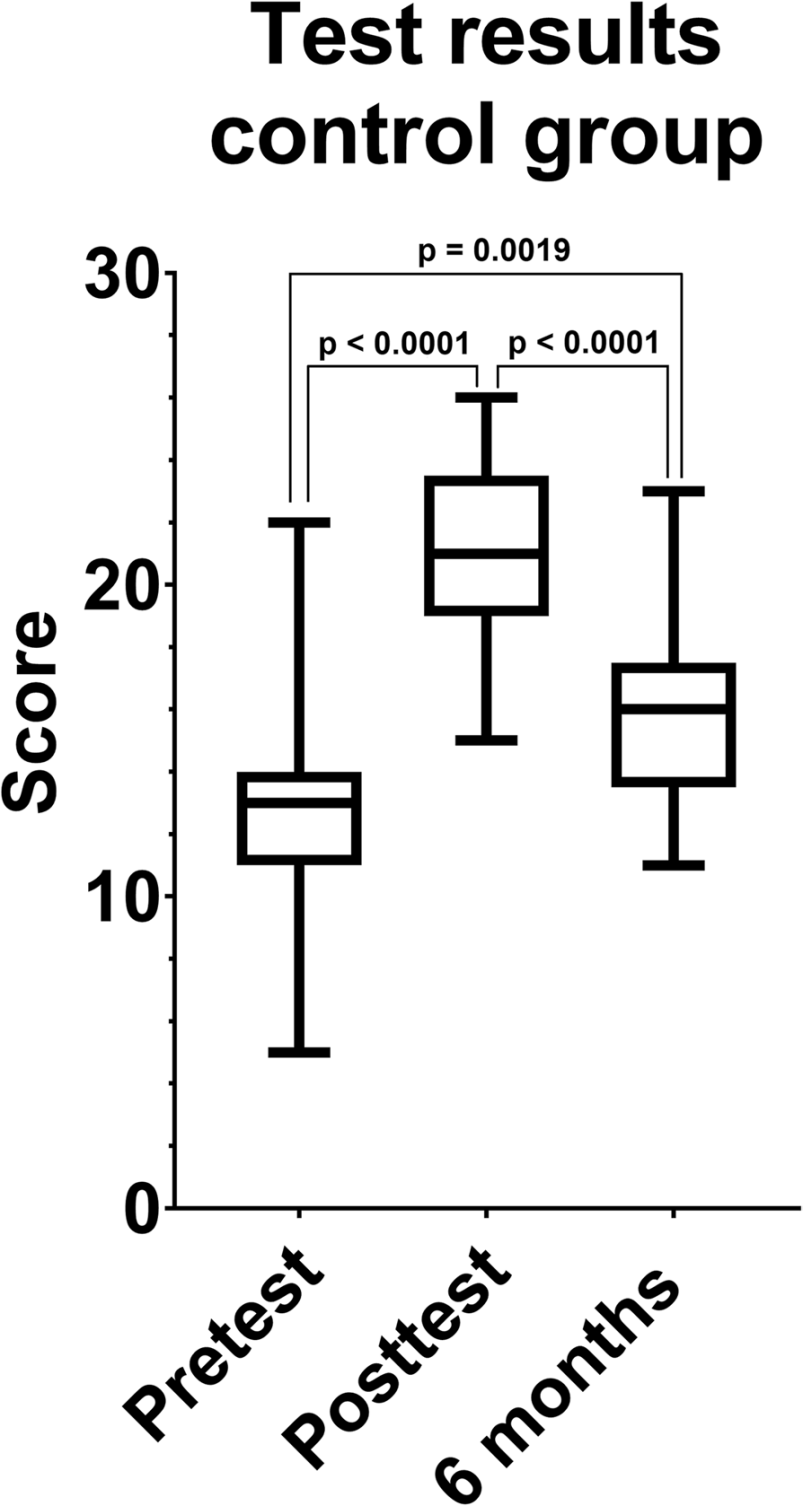
Boxplot comparing the results of the pre-, post- and long-term retention tests in the control group. Participants showed significantly better results in the posttest and long-term retention test compared to the pretest (*p* < 0.0001)

**Fig. 2:**
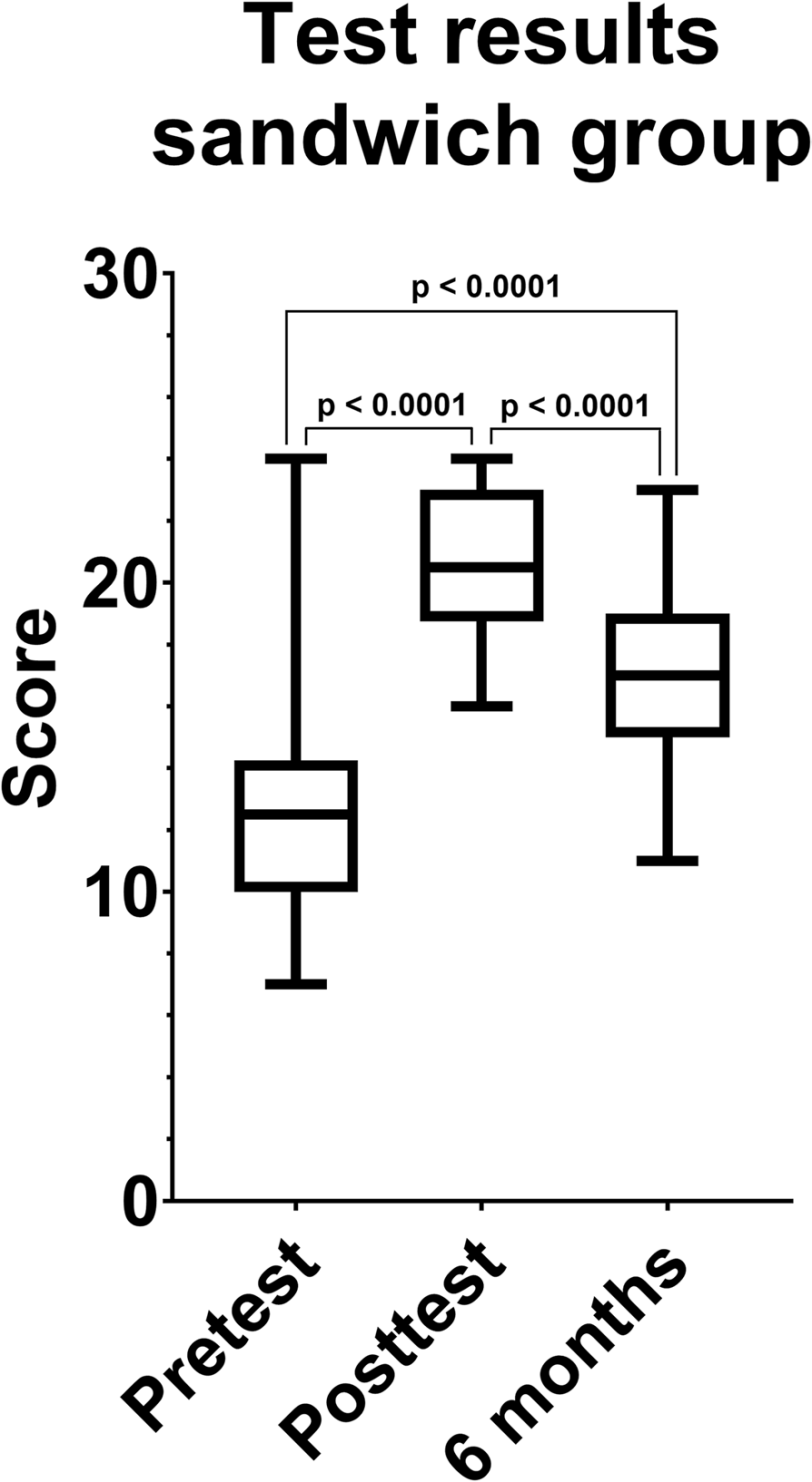
Boxplot comparing the results of the pre-, post- and long-term retention tests in the sandwich group. Participants showed significantly better results in the posttest and long-term retention test compared to the pretest. (p < 0.0001)

Comparing the results of the pretest and the posttest both groups increased their test results significantly (*p* < 0.0001) (Figs. 1 and 2). The control group raised their mean score from 12.6 (SD = 3.52) to 21.28 (SD = 3.11) and the sandwich group from 12.62 (SD = 3.66) to 20.62 (SD = 2.22) points.

Comparing the test results immediately after watching the video and 6 months later, both groups significantly decreased their test results (*p* < 0.0001). The mean score was reduced by 5.33 points in the control group (mean score = 15.95; SD = 3.12) and 3.5 points (mean score = 17.12; SD = 2.91) in the sandwich group. Still, the results of the long-term retention test are significantly better than the results of the pretest (sandwich group: *p* < 0.0001; control group: *p* = 0.0019) (Figs. 1 and 2).

The difference between the groups in the test results of the pretest, the posttest and the long-term retention test was statistically insignificant (*p* = 0.272)) and there was a small effect size (*d* = 0.15). Comparing the results of the posttest and the long-term retention test, it shows a large effect size (d_ppc2_ = 0.847).

### Evaluation

The results of the video evaluation and self-assessment of knowledge acquisition are shown in Table 2. According to the self-assessment, both groups of students significantly improved their knowledge by watching the instructional video (*p* < 0.0001). There was no significant difference in the self-assessment between the two groups (*p* > 0.727) and a small effect size (*d* = 0.15). Evaluating the instructional video itself, both groups rated the video positively. Assuming that the scale midpoint constitutes a neutral rating, there was a significant difference in the sandwich group (*p* < 0.001).

**Table 2:** Results of the evaluation

Aspects of evaluation	Sandwich group mean (SD)	Control group mean (SD)
1. How would you rate the instructional video itself?	3 (1.8)	2.8 (1.6)
2. The instructional video was well structured.	3 (2)	2.1 (1.5)
3. The instructional video conveyed the educational content understandable.	3 (1.5)	2.7 (1.9)
4. My knowledge on cleft lips and palates before watching the instructional video was…	8.6 (2.1)	8.2 (2.1)
5. My knowledge on cleft lips and palates after watching the instructional video was…	4.2 (1.3)	4.1 (1.4)

The sandwich group agreed that the interruptions were useful in helping their understanding of the educational content as they were driven to actively retrieve on previously learned information (mean = 2.29, standard deviation (SD) = 1.67). Moreover, they agreed that the activating elements helped improve their attention and concentration (mean = 3.05, SD = 1.99). They found the interruptions useful, since the previously learned had to be reflected (mean = 2.24, SD = 1.72). The difficulty level of the activating elements was assessed rather difficult (mean = 5.43, SD = 1.4). The group found that the interruptions were placed appropriately throughout the video (mean = 3.91, SD = 2.09). Most students agreed that they could imagine learning in the future using instructional videos that are modified according to the sandwich principle (mean = 2.91, SD = 2.56).

## Discussion

The sandwich principle has previously been applied successfully to lectures. In this teaching model, it has been proven that the application is more effective and leads to a significantly better learning outcome compared with classical lectures [12]. This study intended to investigate whether the application of the sandwich principle to instructional videos leads to improved knowledge gain and how its use is evaluated by students. Therefore, the students’ knowledge was assessed before they watched the instructional video and immediately after the lecture to assess their short-term retention of information. Six months later, a written test to evaluate the long-term recall of knowledge took place. For all three tests, the same test questions were used. Additionally, the students’ satisfaction with the instructional video and the modification was assessed. This study shows that the hypothesis of modified instructional videos according to the sandwich principle lead to an improved learning outcome could not be proven subjectively and objectively.

A comparison of the results of the posttest and long-term retention test to the baseline level of knowledge revealed that both groups had significantly better results after watching the instructional video both times. When only the results of the posttest and the long-term retention test were compared, both groups showed a decrease in their mean score. Although this decrease was significant in both groups, the results of the sandwich group were slightly better than those of the control group. This outcome might indicate a beneficial long-term effect for the modified instructional video owing to the activating elements. This long-term effect could be analysed better through with an increased testing scope, a different testing format or a different choice of interval for testing long-term retention [18].

.In addition, there was no significant difference in the test scores between the sandwich and control groups. Therefore, the interruptions for the activating elements did not induce the desired testing effect, retrieval practice to boost the long-term learning [19]. Multiple previous studies have shown beneficial long-term learning in the context of retrieval practice [20–22]. In this study, the testing effect may have been attributed to the posttest in which both groups participated and therefore, both groups had equally good results.

The results of the self-assessment of knowledge acquisition likewise show that the students assessed their own knowledge equally good before and after watching the instructional video. Therefore, the objective and subjective results enable us to assume that the instructional video itself must have a good teaching effect. This notion is confirmed by the evaluation of both groups, who verified the advantageous didactic effect of the video itself. The hypothesis that the modification of instructional videos according to the sandwich principle leads to an improved learning outcome could not be proven. Therefore, whether the extra work of developing activating elements and editing the video for the modification is worth the effort must be discussed.

The evaluation showed that the students in the sandwich group highly appreciated the modified video. They found the interruptions useful for repetition of previously learned information. The participants also confirmed that the interruptions improved their concentration and attention to the video. They pointed out that they would like to learn in the future using instructional videos modified according to the sandwich principle. In general, the use of multimedia is highly appreciated by students and can be a powerful supplement and motivator to classical teaching formats [23–25]. A review by Green et al. found improved knowledge, skills performance and learner satisfaction using video-based training resources compared with non-video training groups [26]. This finding allows us to conclude that, from the students’ point of view, the modified teaching format is effective and indicated.

From the teachers’ point of view, applying the sandwich principle to instructional videos requires a great deal of work. Besides setting a special focus when establishing the learning objectives, the activating elements have to be created and integrated into the video. These preparations are time consuming, especially under the aspect of an equal beneficial learning outcome. Nevertheless, once created, the modified instructional video is a sustainable teaching method that offers students a flexible, asynchronous study method by being independent of time, place and speed [27–30]. Although video production costs are high in the beginning, after several years of usage, digital learning has been shown to have lower costs due to the reduced need for institutional infrastructure and resources [31].

.In the present study, the length of the instructional video (45 min) can be regarded as a limitation. In general, shorter instructional videos are better for the attention span. Shell et al. found that the optimal length of instructional videos is 5 - 10 min [32]. According to Bunce et al. the attention span is 20-25 min in class, so that in this study the attention span was considered in the sandwich group by placing the activating elements after 15 - 20 min [8]. The length of the instructional video in this study can also be seen as an advantage because it provides better comparability of the effects of lectures. Another important limitation of this study is that the duration of learning phase was longer in sandwich group than in the control group due to the activating elements. This was unavoidable as both groups were supposed to watch exactly the same instructional video. In this study, the test can be seen as another limitation, as all three tests used the same test questions. The pretest may have guided learners’s attention to the requested information so that in both groups’ participants paid more attention to the information they had not known. Such a viewing behaviour may cover potential differences between the two groups. Therefore, in future studies different test questions should be used.

## Conclusion

The hypothesis that the modification of instructional videos according to the sandwich principle leads to an improved learning outcome could not be proven subjectively and objectively. Nevertheless, the teaching format is highly appreciated by the students and may increase their motivation to learn with instructional videos. Therefore, instructional videos modified according to the sandwich principle are a possible option when transitioning from traditional to digital teaching.

## Supplementary Information

**Additional file 1.**

## Data Availability

The datasets used and/or analysed during the current study are available from the corresponding author on reasonable request.
