# Correction to: Transferring the sandwich principle to instructional videos: is it worth the effort?

**DOI:** 10.1186/s12909-022-03103-5

**Published:** 2022-01-07

**Authors:** Anna Bock, Christina Thomas, Marius Heitzer, Philipp Winnand, Florian Peters, Martin Lemos, Frank Hölzle, Ali Modabber

**Affiliations:** 1 Department of Oral and Maxillofacial Surgery, University Hospital RWTH Aachen, Pauwelsstrasse 30, 52074 Aachen, Germany; 2 Audiovisual Media Center, Medical Faculty, RWTH Aachen University, Pauwelsstraße 30, 52074 Aachen, Germany

**Correction to: BMC Med Educ 21, 525 (2021)**

**https://doi.org/10.1186/s12909-021-02967-3**

Following publication of the original article [1], we have been informed that the given name and family name were erroneously transposed for all the authors.

The author group has been updated above and the original article [1] has been corrected.
