# Supplementary material for: Transferring the sandwich principle to instructional videos: is it worth the effort?
Source: BMC Med Educ. 2021 Oct 9;21:525. doi: 10.1186/s12909-021-02967-3 (PMC8501714; doi:10.1186/s12909-021-02967-3)
Supplement: Supplementary file 1 — Additional file 1. [file 12909_2021_2967_MOESM1_ESM.docx]

**Evaluation questionnaire**

1. How would you rate the instructional video itself?

□ □ □ □ □ □ □ □ □ □

1 2 3 4 5 6 7 8 9 10

very good unsatisfactory

1. The instructional video was well structured.

□ □ □ □ □ □ □ □ □ □

1 2 3 4 5 6 7 8 9 10

fully agree totally disagree

1. The instructional video conveyed the educational content understandable.

□ □ □ □ □ □ □ □ □ □

1 2 3 4 5 6 7 8 9 10

fully agree totally disagree

1. My knowledge on cleft lips and palates before watching the tutorial video was…

□ □ □ □ □ □ □ □ □ □

1 2 3 4 5 6 7 8 9 10

very good unsatisfactory

1. My knowledge on cleft lips and palates after watching the tutorial video was…

□ □ □ □ □ □ □ □ □ □

1 2 3 4 5 6 7 8 9 10

very good unsatisfactory

**Evaluation of activating elements (only Sandwich-Group)**

1. The interruptions during the video for the activating elements were useful in helping my understanding of the educational content.

□ □ □ □ □ □ □ □ □ □

1 2 3 4 5 6 7 8 9 10

fully agree totally disagree

1. The activating elements helped improve their attention and concentration.

□ □ □ □ □ □ □ □ □ □

1 2 3 4 5 6 7 8 9 10

fully agree totally disagree

1. The interruptions were useful, since the previously learned had to be reflected.

□ □ □ □ □ □ □ □ □ □

1 2 3 4 5 6 7 8 9 10

fully agree totally disagree

1. How would you rate the difficulty level of the activating elements?

□ □ □ □ □ □ □ □ □ □

1 2 3 4 5 6 7 8 9 10

Too easy too difficult

1. How were the interruptions placed throughout the video?

□ □ □ □ □ □ □ □ □

1 2 3 4 5 6 7 8 9 10

appropriate inappropriate

1. I could imagine learning with instructional videos modified according to the sandwich principle in the future.

□ □ □ □ □ □ □ □ □ □

1 2 3 4 5 6 7 8 9 10

fully agree totally disagree
